# Long-term follow-up of patients treated with multiple fluocinolone acetonide implants for noninfectious uveitis

**DOI:** 10.1007/s12348-012-0064-z

**Published:** 2012-03-13

**Authors:** Rebekah C. Allen, Eric B. Suhler, Christina J. Flaxel, Zunqiu Chen, Dongseok Choi

**Affiliations:** 1 Casey Eye Institute, Oregon Health & Science University, 3375 SW Terwilliger Blvd., Portland, OR 97239 USA; 2 Portland Veterans Affairs Medical Center, Portland, OR USA; 3 Department of Public Health & Preventive Medicine, Oregon Health & Science University, Portland, OR USA

**Keywords:** Anti-inflammatory agents, Drug implants, Fluocinolone acetonide, Uveitis/drug therapy, Visual acuity/drug effects

## Abstract

**Purpose:**

To evaluate long-term outcomes in eyes undergoing exchange of fluocinolone acetonide intravitreal implants for noninfectious uveitis.

**Methods:**

In this retrospective case series, chart review was conducted of all patients treated for noninfectious uveitis with fluocinolone acetonide implants. All patients were seen at a single center between 2007 and 2010.We studied eight eyes of eight patients who received second implants in exchange for previously placed implants and received follow-up care after the implant was exchanged. Main outcome measures were visual acuity (VA), recurrence of inflammation, need for adjunctive systemic anti-inflammatory treatment and adverse events.

**Results:**

We studied eight eyes of eight patients. Average length of follow-up after the second implant was 32.3 months. Of the eight patients, five experienced improvement or stabilization of VA when acuity prior to the initial implant was compared to acuity on long-term follow-up. After their first implant, five patients experienced disease recurrence. Including all eight patients, the estimated median time to recurrence was 35.7 months after the first implant. The mean time to reimplantation was 42.7 months. After the second implant, three patients experienced recurrence. Including all eight patients, the estimated median time to recurrence was 30.1 months after the second implant. Adverse events included perioperative complications, elevated intraocular pressure (IOP) and cataracts.

**Conclusions:**

Exchanging FA intravitreal implants used to treat noninfectious uveitis may be useful in preventing vision loss and recurrence of inflammation. Development of elevated IOP and cataract is a potentially serious complication. The risks and benefits of implant exchange must be carefully considered with this intervention.

## Introduction

Chronic noninfectious uveitis is a potentially blinding condition [1, 2]. Control of inflammation is imperative to ameliorate the individual and socioeconomic impacts of vision loss from this disorder [3]. Treatment often requires the use of local and systemic corticosteroids and noncorticosteroid systemic medications. However, the potential side effects of systemic medications require regular monitoring [4]. Side effects are also associated with repeated topical, periocular and intravitreal corticosteroid treatment [5]. The fluocinolone acetonide implant was developed in an attempt to decrease the risks associated with systemic side effects and the need for recurrent local corticosteroid treatment. It was approved by the US Food and Drug Administration for this use in 2005. The implant is designed to release a controlled amount of fluocinolone acetonide into the eye for a 30-month period. The original study evaluated the safety and efficacy of the original fluocinolone acetonide implant for 3 years (36 months) after implantation [6]. The corticosteroid reservoir is believed to be depleted in 2.5 to 3 years (30 to 36 months); thus far, two follow-up studies have evaluated the safety and efficacy of implant replacement [7, 8]. Average follow-up time in these studies was 17 months. In this study, we aim to characterize longer-term outcomes in the eyes of patients with noninfectious uveitis who undergo exchange of fluocinolone acetonide implants.

## Materials and methods

This single-center, retrospective case series was approved by the institutional review board of Oregon Health & Science University. Patients were identified through billing codes for surgical intravitreal implants placed between January 2007 and October 2010 at the Casey Eye Institute, Oregon Health & Science University. Inclusion criteria were a diagnosis of recurrent noninfectious uveitis, the exchange of a fluocinolone acetonide implant and at least 1 month of follow-up after the second implant was placed. Of the patients who underwent placement of a fluocinolone acetonide implant, eight eyes of eight patients had exchange of the implant and met the inclusion criteria. Of these eight patients, two patients went on to receive their third implants. We reviewed the charts of all eight patients in detail.

The exchange of the intravitreal implants with 0.59-mg fluocinolone implants was performed in the operating room by a vitreoretinal surgeon after written informed consent was obtained. Reimplantation was performed according to previously described procedures [7, 9]. Each case was left to the discretion of the operating surgeon as to whether the original implant was removed and whether the new implant was placed in the original site or moved to a new site. Overall, two implants were removed with placement of the subsequent implant in the same site, and the remainder of the implants were placed in new sites. All patients were treated with topical corticosteroids postoperatively and with oral corticosteroids or other systemic immune system modulators at the discretion of the surgeon and the uveitis specialist.

The outcome measures assessed were visual acuity (VA), recurrence of inflammation, perioperative complications and adverse events. VA was measured by a Snellen chart before and after the first, second and, in some cases, third implants. The best-corrected VA was recorded where available; at visits where no manifest refraction was performed, the pinhole acuity was recorded. Recurrence of inflammation was defined as one of the following: 1) increase of greater than or equal to two steps in the number of cells in the anterior chamber compared to baseline, 2) increase of greater than or equal to two steps in vitreous haze compared to baseline, 3) decrease of ≥0.30 logMAR VA compared to baseline with evidence of either cystoid macular edema (CME) or active inflammation or 4) development of CME due to inflammation [6]. In conjunction with recurrence of inflammation, we assessed the need for systemic corticosteroid or corticosteroid-sparing medications before the first implant and after the first and subsequent implants. We also assessed adverse events including perioperative complications, device-specific complications, the development of elevated IOP and the formation of visually significant cataracts. Perioperative complications were defined as occurring within 90 days of surgery and specific to the surgical procedure.

Patient demographics were assessed and included gender, diagnosis, age at first implant, time to second implant, time to third implant and total days of follow-up. We compared VA preimplant, postfirst implant and postsecond implant using the paired *t* test. Following placement of the first implants, data were collected at time points ranging from 24 to 36 months postimplant. Following placement of the second implants, data were also collected at a wide range of time points, from 16.7 to 43.1 months postsecond implant. Kaplan–Meier estimates were performed to determine the time to recurrence after the first, second and third implants. The development of elevated IOP and cataracts was calculated using the incidence density.

## Results

Eight eyes of eight patients were studied, with demographic data summarized in Table 1. The average age at first implant was 40.6 years and 44 years at second implant. The average time to second implant was 42.7 months. Implant exchange was recommended based on recurrence of inflammation that subsided with local or systemic corticosteroid treatment or on the desire to continue suppressing inflammation when the implant was theoretically depleted based on prior data (6). The average time from second to third implant was 30.2 months for the two patients receiving a third implant. The average length of follow-up after the second implant was 32.3 months (range 16.7–43.1 months) and 19.5 months after the third implant (range 5.8–33.2 months).

**Table 1:** Demographic information for eight patients receiving exchange of fluocinolone acetonide implants (Retisert) for management of noninfectious uveitis

Patient no. (eye)	Gender	Age at 1st implant	Ocular diagnosis	Systemic involvement	Other eye involvement
1	F	25	Multifocal choroiditis	No	Yes
2	F	45	Sarcoidosis	Yes	Yes
3	F	41	Multifocal choroiditis	Yes	Yes
4	M	41	HLA-B27 positive uveitis	Yes	No
5	M	39	Serpiginous choroiditis	No	Yes
6	F	63	Birdshot choroidopathy	No	Yes
7	F	35	Idiopathic panuveitis	No	Yes
8	M	36	HLA-B27 positive uveitis	Yes	No

Final VA comparisons are presented in Table 2. Four of the eight patients experienced improved VA based on preoperative and final follow-up acuity measurements. One patient maintained stable acuity. Three patients had worsened VA at the final follow-up compared to initial acuity. Of these, one patient had intractable hypotony maculopathy 5.6 months after the second implant, one patient developed a subfoveal choroidal neovascularization after the first implant and one patient had recurrent ocular inflammation despite adjunctive treatment. VA comparisons before and after the first implant, before the first and after the second implants, and after the first and after the second implants were similar, with a 95 % confidence interval (−0.1520, 0.3683), (−0.2626, 0.1992), (−0.3177, 0.1338).

**Table 2:** Visual acuities of seven patients receiving exchange of fluocinolone acetonide implants (Retisert) for management of noninfectious uveitis

	Number	Mean difference	Standard deviation	95 % CL standard deviation
Preimplant versus postfirst implant	7	0.1081	0.2813	(0.1813, 0.6194)
Preimplant versus postsecond implant	7	−0.0317	0.2497	(0.1609, 0.5497)

**Fig. 1:**
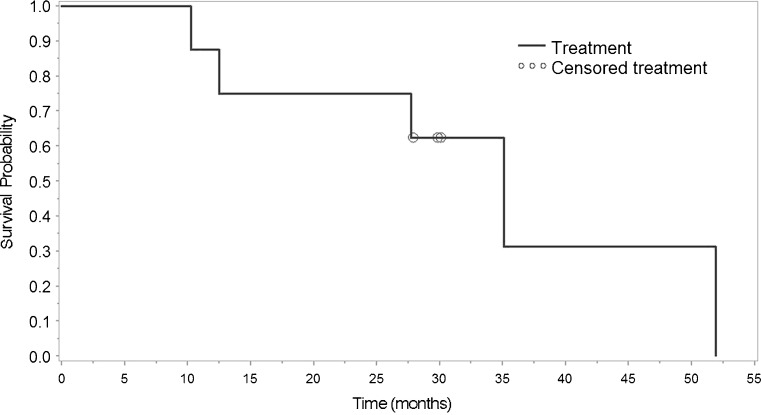
Kaplan–Meier analysis of time to recurrence following implantation of a fluocinolone acetonide implant in eight patients treated for noninfectious uveitis

**Fig. 2:**
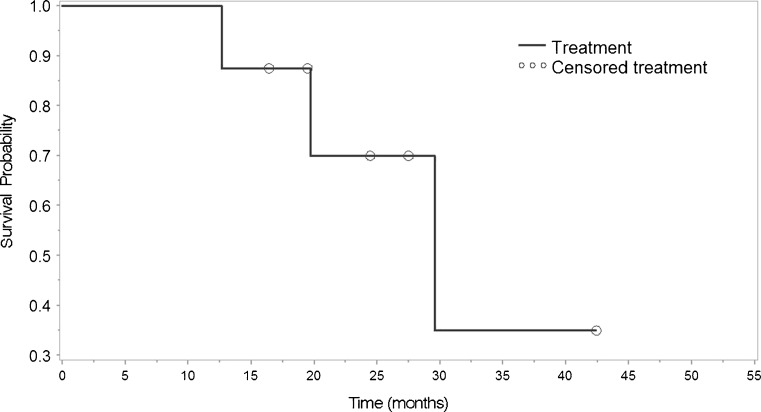
Kaplan–Meier analysis of time to recurrence of noninfectious uveitis in eight patients following reimplantation of fluocinolone acetonide implant

During the 52 weeks prior to receiving the initial fluocinolone implant, all patients had at least one recurrence of inflammation. After the first implantation, five of the eight patients experienced a recurrence; however, in one of these five, the recurrence occurred 1 month after the first implant was removed due to extrusion of the implant, with implant removal 49.2 months after placement. After the second implant, three patients had recurrence of inflammation. When comparing the first and second implants, the median time to first inflammation recurrence was 35.7 months for the first implant and 30.1 months for the second implant. The Kaplan–Meier curves for these data are in Figs. 1 and 2. Of the two patients who received a third implant, the first patient received it secondary to recurrence of inflammation and the second received it secondary to a desire to prevent recurrence of inflammation when the implant depleted.

Six of the eight patients were receiving systemic immunosuppressive therapy before the first implant. One patient’s prior immunosuppression status was not known. One patient never required immunosuppression throughout the disease process. After the first implant, three of the six patients taking systemic medication were able to completely taper off immunosuppressive therapy and did not require it again through the final follow-up visit. One patient remained on systemic immunosuppression; however, it was used for joint pain and never indicated for ocular inflammation. The remaining two patients required two different systemic immunosuppressive medications prior to the first implant and only one systemic medication after both the first and second implants.

Adverse events included perioperative complications, device-related long-term complications, development of elevated IOP and formation of visually significant cataracts. Perioperative complications occurred after both the first and second implants. After the first implant, one patient had perioperative vitreous hemorrhage. Following the second implant, one patient had an exposed scleral wound requiring a scleral patch graft and another patient had a vitreous hemorrhage. Intraoperatively, one patient had a suprachoroidal hemorrhage and recurrent retinal detachments during placement of the third implant, and one patient had conjunctival dehiscence at the wound site. The only long-term device-related complication was extrusion of the first implant in one patient 49.2 months after placement. The implant was removed, and the patient experienced recurrent inflammation the following month. A second implant was placed. A total of six patients developed elevated IOP after implantation, necessitating four glaucoma surgeries and two laser procedures in three eyes after the first implant and three surgeries in three eyes following the second implant. No surgeries were required following the third implant. After the first implant, four of four phakic eyes developed visually significant cataracts requiring surgery.

## Discussion

Chronic noninfectious uveitis is a potentially blinding disease, with or without treatment. This retrospective case series demonstrates that exchanging fluocinolone acetonide intravitreal implants may help prevent vision loss and recurrent inflammation.

VA was improved or maintained in five of the eight patients studied (62.5 %) and decreased in three patients (37.5 %) over the course of the treatment. In the three patients whose VA worsened, one had previously worsened acuity secondary to hypotony maculopathy from an overfiltering glaucoma valve. The remaining two patients had worsened acuity secondary to subfoveal choroidal neovascularization and recurrent inflammation, respectively. These problems were more likely related to the underlying disease process and not the implant itself. In all three cases, vision loss worsened despite adjunctive local or systemic immunosuppression or both. Overall, the comparison of VA prior to implantation and at final follow-up was not statistically significant in this small patient population.

In the two prior studies of implant exchange, VA stabilized or improved in 100 % of patients [7, 8]. However, both studies had an average follow-up time of 17 months after the second implant. Our study had an average follow-up time of 32 months after the second implant. The median implant survival time prior to recurrence was similar between the first and second implants, at 35.7 and 30.1 months, respectively. This is similar to the data from the two prior studies of replacement implants [7, 8]. After the third implant, median survival time until recurrence was only 8 months; however, just two patients received a third implant. Of these, only one patient experienced recurrent inflammation. This patient had severe recurrent inflammation with all three implants and was never able to discontinue systemic corticosteroid therapy despite adjunctive local and systemic treatment in addition to the implants. Longer follow-up may reveal more VA loss, given the chronic nature of noninfectious uveitis.

Past data suggest that the implant itself may have a poorly understood mechanism for extended therapeutic effect. Thus, it is unknown if exchanging a theoretically depleted implant in a quiescent eye is helpful or if the inflammation would have remained quiescent despite the intervention. In all six patients who required systemic immunosuppressive medications before the first implant, all were able to eliminate or reduce the number of medications needed to control the inflammation. This is similar to the findings of the two prior studies of implant replacement [7, 8].

Adverse effects included perioperative complications, long-term device related complications and the development of cataracts and elevated IOP. In most cases, the perioperative complications were easily treated, except in the patient with suprachoroidal hemorrhage and subsequent recurrent retinal detachments. This is consistent with prior data regarding the implants. The single long-term device-related complication, implant extrusion, occurred 4 years after the initial implant and responded well to implant exchange with no permanent deleterious effects. The risk of perioperative side effects was similar when comparing the first and second implants. The development of cataracts occurred during the first implant and was not a side effect of the second implant, as the patients had already undergone cataract surgery prior to the second implant placement. The risk of glaucoma surgery was similar after the first and second implants (three patients required surgery after each implant); however, the number of surgeries required by the three patients was six after the first implant (including laser surgery), and the number required by the three patients after the second implant was three surgeries. The development of visually significant cataracts and elevated IOP is associated not only with chronic recurrent uveitis but is also a known complication of the fluocinolone implant [6]. A recent therapy trial demonstrated favorable outcomes for placement of a glaucoma tube shunt in conjunction with a first implant in eyes that already had elevated intraocular pressures (IOPs) on maximal medical therapy trial [10]. Given the expected high rates of elevated IOP requiring surgery, clinicians might want to consider this therapeutic option for future patients.

The current study has several limitations. This is a single-center retrospective study with a small number of patients. There is also the possibility of selection bias, in that a group of patients undergoing multiple intravitreal implants is more likely to include those patients who responded well to this approach initially. It might be expected that the complication rate in patients undergoing a second or third implant might be less, as this group of patients may have some physiological reasons for lower complication rates that are not readily identifiable. Regression to the mean is another limitation of our study, in that there is a tendency for patients with multiple recurrences leading up to the intravitreal implant to have fewer recurrences over time. This could be due to the natural course of the disease and not necessarily to the implant itself. In addition, this study was not designed to evaluate and compare findings from each patient’s fellow eye to determine if the inflammation had naturally run its course regardless of intravitreal implant placement and exchange. Results of the recently published Multicenter Uveitis Steroid Treatment (MUST) Trial [11] indicated that VA and inflammation control improved over 24 months in patients receiving both systemic treatment and intravitreal implants. There was no clear evidence favoring either approach. The study concluded that selection of treatment method should be dictated by the individual patient’s particular circumstances.

## Conclusion

Exchanging a depleted fluocinolone acetonide implant is a viable treatment for controlling intraocular inflammation caused by chronic noninfectious uveitis. This treatment may be helpful in preserving VA and potentially quality of life, though our study did not directly assess quality of life. It may also reduce the need for systemic corticosteroids or corticosteroid-sparing medications, with their associated side effects. The adverse effects of perioperative complications, development of cataract and development of elevated IOP are significant and have been documented in previous studies. Given the natural history of noninfectious uveitis and the significant risks of treatment complications from this therapy, the risks and benefits must be carefully considered for each patient. MUST Trial results have suggested that systemic adverse effects of systemic therapy were not very frequent; thus, the clinician and patient must weigh the potential risks of subsequent intravitreal implants.
